# Tannins: Prospectives and Actual Industrial Applications

**DOI:** 10.3390/biom9080344

**Published:** 2019-08-05

**Authors:** Antonio Pizzi

**Affiliations:** LERMAB-ENSTIB, University of Lorraine, 27 rue Philippe Seguin, 88000 Epinal, France; antonio.pizzi@univ-lorraine.fr

**Keywords:** tannins, applications, new applications, drawbacks, advantages

## Abstract

The origin of tannins, their historical evolution, their different types, and their applications are described. Old and established applications are described, as well as the future applications which are being developed at present and that promise to have an industrial impact in the future. The chemistry of some of these applications is discussed where it is essential to understand the tannins and their derivates role. The essential points of each application, their drawbacks, and their chance of industrial application are briefly discussed. The article presents historical applications of tannins, such as leather, or traditional medicine, and more recent applications.

## 1. Origin of Tannins

Leather tanning has been used for centuries, even millennia, by immersing skins in water where special barks or woods containing tannin have been added. Up to a full year was necessary for leather to be produced in such a manner. However, the current tannin extraction industry is relatively newer. The more modern history of tannins began in the 17th century when Giovannetti, an Italian chemist, studied the interactions between iron solutions and substances called “astringents”. In 1772, various researchers identified the presence of an acid in these compounds. This acid was then isolated by Scheele and turned out to be gallic acid. Based on the experiments of Deyeux and Bartholdi, continued by Proust in the late 18th and early 19th centuries, tannins have been officially recognized as a discrete group of different molecules based on gallic acid content. The great growth of the tannin extraction industry began in the years around 1850 in Lyon, where tannin was used as iron tannate for the black coloring of silk for women’s blouses [1]. After 10 years, fashion changed and thus after many bankruptcies and groupings of factories, tannin manufacturers were able to convince the leather industry to use tannin in place of oak chips with considerable savings in tanning time (from 12 months with the old system based on wood chips rich in tannin to 28 days using tannin extract) [1]. The benefits of tannin extracts in the manufacture of leather, and even the time savings still allowed by their use, were such that the industry expanded rapidly and thrived. Tannin being in short supply in Europe, factories were opened in distant countries to satisfy the growing demand and promoting the use of alternative tannin types. Early in the 20th century, South American and southern and central African tannins began to be industrially extracted to supply major markets in Europe and North America. Among these, the main ones were quebracho wood and mimosa bark tannins. Leather processing was thus the second major boom period for the tannin industry. After World War II the substitution of leather with synthetic materials for shoes again caused a number of tannin extraction plants to close [2]. The third period of use of tannin therefore began, first with their development as bio-based adhesives and later with an increasing number of applications in new bio-based materials, this latter period being still in full development.

The name “tannin” comes from the use of this class of compounds in the tanning process of hides to give leather. Their general appearance varies, ranging from white amorphous powders to off-white amorphous powders, to glossy, almost colorless pasty substances, to reddish-brown powders when produced by spray drying. They have an astringent taste. Tannins are natural products found in most higher plants. They are produced in almost all parts of the plant, namely seeds, roots, bark, wood, and leaves, because of their fundamental role in the defense of the plant against insects, food infections, fungi, or bacteria. The defense mechanism is based on the ability of tannins to complex proteins irreversibly. They are also considered as one of the effective components contributing to the fact that the risk of suffering from cardiovascular diseases and some forms of cancer can be reduced by choosing diets rich in fruits and vegetables. In addition to their documented effects on human health, tannins are also important for the welfare of ruminants; high protein feeds such as alfalfa trigger the production in the rumen of methane trapped as proteinaceous foam, resulting in a potentially mortal fermentation that can be reduced by adding tannin in the diet. Two wide classes of tannins exist: hydrolysable tannins such as gallo-tannins and ellagi-tannins, and condensed polyflavonoid tannins, these latter being stable and rarely subject to hydrolysis [2].

### 1.1. Hydrolysable Tannins

The hydrolysable tannins, usually present in small amounts in plants, are simple derivatives of gallic acid, and they are classified according to the products obtained after hydrolysis—gallo-tannins (gallic acid compounds and glucose) and ellagi-tannins (composed of biaryl units and glucose).

**Figure 1:**
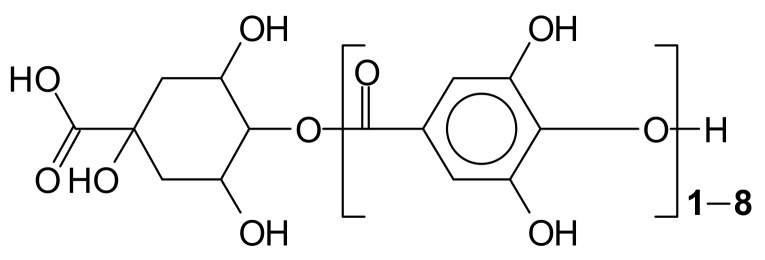
Example of the structures of tara tannin [3,4] and caffe-tannins (quinic acid esterified with caffeic acids plus glucose compounds).

Most gallo-tannins isolated from plants contain a polyol residue derived from D-glucose, although a large variety of polyol types can be found. Two other categories, gallo-tannins of tara (composed of gallic and quinic acid and glucose) and caffe-tannins (quinic acid esterified with caffeic acids plus glucose compounds), also occur [3,4] (Figure 1).

#### 1.1.1. Gallo-tannins

Tannic acid is one of the most important substances in relation to hydrolysable tannins. Tannic acid exists de facto in the form of a mixture of very similar substances, for example penta-(digalloyl)-glucose and tetra-(digalloyl)-glucose or tri-(digalloyl)-di-(galloyl)-glucose, etc. [4,5,6].

#### 1.1.2. Ellagi-tannins

**Figure 2:**
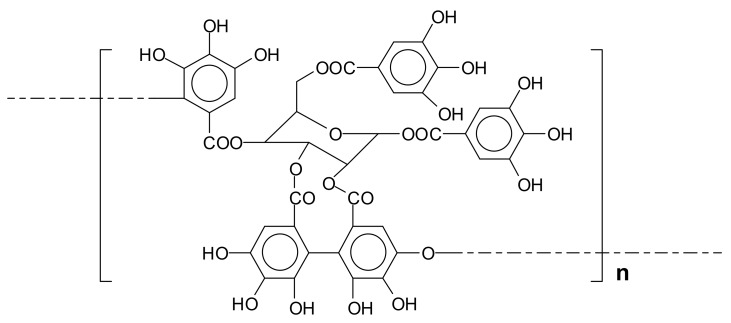
Schematic representation of the repeating unit of the ellagi-tannin of chestnut wood.

Different from gallo-tannins, ellagi-tannins contain additional binding motifs that arise from additional oxidative coupling reactions between the galloyl fragments [6]. The biosynthesis of ellagi-tannin is therefore an oxidative enzymatic progression of gallo-tannins. The first step is an oxidation of 1,2,3,4,6-pentagalloylglucose to form the monomeric ellagi-tannin. The second step consists essentially of dimerization after a second enzyme-mediated specific oxidation of hexahydroxydiphenoyl group of the ellagi-tannin with the galloyl group of another tannin to form a valoneyl group-containing dimer and these reactions continue to form higher oligomers. An example of ellagi-tannin is the tannin of chestnut wood (Figure 2).

### 1.2. Condensed Polyflavonoid Tannins

**Figure 3:**
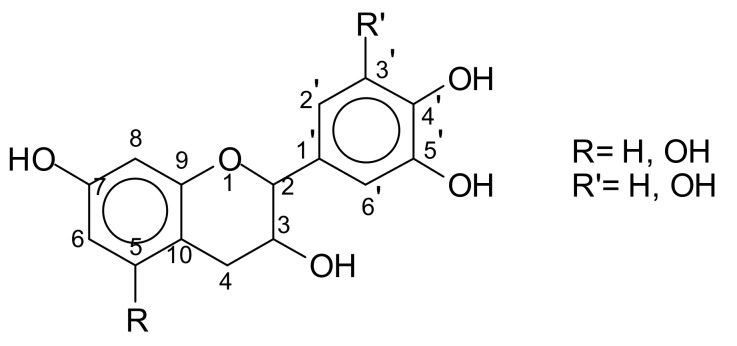
The structure of a flavonoid unit.

Condensed tannin extracts consist of flavonoid oligomers of different average degrees of polymerisation. Small proportions of flavan-3-ols, flavan-3,4-diols, and other flavonoid analogs are also present [7,8]. Carbohydrates, such as broken down residues of hemicelluloses, and hexoses, pentoses, and disaccharides together with some imino acids and amino acids [2,9] constitute the non-phenolic part of tannin extracts. These latter, as well as the monoflavonoids, are equally present in too low a proportion to influence the extract properties. On the contrary, oligomers derived from hydrolysed hemicelluloses are often present in sufficient quantities. Equally, carbohydrate chains of various lengths [4,5] are also sometime linked to the flavonoid unit in the tannin. The basic structure of these tannins is based on the flavonoid unit (Figure 3).

These flavonoid units are generally linked C4 to C6, or C4 to C8 to forms a variety of short chains of different lengths according to the type of tannin.

## 2. Industrial Utilization

The industrial uses briefly described below are in order of present or probable future importance to give an idea of what is developed and used already, and which applications are likely to gather importance in the future.

### 2.1. Leather Tanning

The manufacture of leather is still the largest use of tannins of vegetable origin. Leather has traditionally been made in ground pits in which alternating layers of animal skins and wood chips containing tannins, such as oak chips, have been placed and soaked for considerable periods of time. The skins were passed through a number of consecutive pits, generally 28, so as to be slowly enriched and “tanned” by the tannin in solution. The tannins exfiltrated the wood chips and slowly impregnated increasingly more of each skin. Such a manufacturing system was practiced for many centuries and produced good quality leather but it took several months, often a whole year, for the leather to be ready. The first change came when the tannin extraction industry, which had grown considerably in the 1850s to supply black iron tannate dyes to color silk, was in a desperate position because of the change in fashion. Some tannin extraction plants were able to demonstrate that by directly adding tannin extract to traditional tanning pits, the same quality of leather could be produced in just 28 days (28 pits, one per day). Leather tanned in this way began to prevail and this use led to a boom which lasted mainly until the end of the Second World War. In particular, the war years were good simply because armies were walking in leather boots.

At the end of the Second World War, three events contributed significantly to the steady decline in vegetable tanned leather. First, the introduction of synthetic materials, derived from petrol, for shoe soles begun to compete strongly with leather for one of its most traditional applications. Secondly, the demobilization of armies, which sharply reduced the need for leather shoes, and the third, the strong penetration of the market by chromium salt tanning for the manufacture of soft leathers, in particular the upper part of shoes. With all these changes, while vegetable leather has now begun to gain a reputation as a luxury product, there are still some important niches for which it is used such as equestrian equipment, heavy bags and luggage, and other heavy applications as well as the real high price luxury markets. Although the traditional 28 pits tanning method still exists for a number of special leathers as well as in the case of artisanal leather (as in Morocco for example), the tanning processes has also evolved for vegetable tanning where rotary drums, a technique borrowed from chrome tanning, allows vegetable tanning to be finalized in about 24 h. Today, research on vegetable tanned leather has been able to produce much more supple leather through the inclusion of oils and other techniques, so that some rebirth of the use of plant tannins for other application areas appears to take place.

### 2.2. Wood Adhesives

There are a number of detailed reviews on the use of tannins for wood adhesives. The reader is referred to these detailed studies [2,10]. However, here existing technologies and industrial use of wood tannin adhesives are presented.

**Figure 4:**
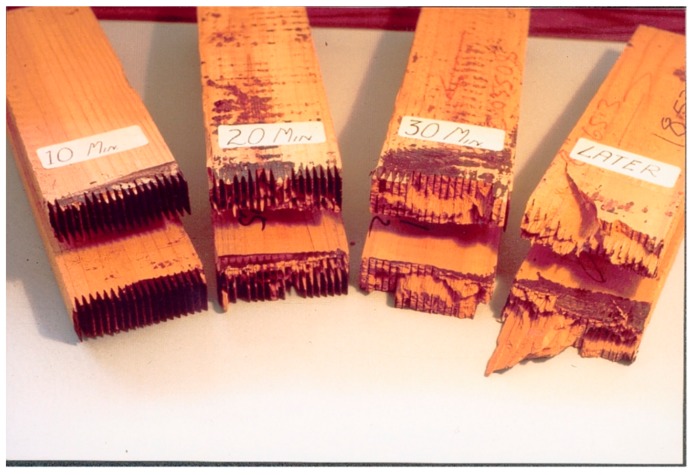
Development of strength visualized through the rapid increase in percentage wood failure of tannin-based fast-setting “honeymoon” separate application cold-setting adhesives for glulam and fingerjoints.

As extensive studies already exist, and this application of tannin is now the second most important after leather manufacturing, only a few of the main achievements of tannin-based adhesives for wood products will be highlighted. (1) The development, optimization, and industrialization of non-fortified but chemically modified thermosetting tannins for particleboard, other particle products, and plywood [11,12,13]. (2) The technology for rapidly pressing tannin adhesives for particle board, which is also industrial [14,15]. (3) The development and industrialization of tannin–urea–formaldehyde adhesives for plywood and in particular as impregnators for corrugated board starch binders [16]. (4) The development and industrialization of cold-setting tannin–resorcinol–formaldehyde adhesives for glulam and fingerjointing [17]. (5) The large-scale development and industrialization of fast-setting “honeymoon” separate application cold-setting adhesives for tannin-bonded glulam and fingerjoints [18,19,20] (Figure 4).

(6) The development and industrialization of zinc salts to accelerate the hardening of non-fortified tannin adhesives for plywood [2,21,22,23]. (7) Successful formulation, development, and industrialization in Chile of pine bark tannin adhesives for particle boards and for glulam and fingerjointing [13,24]. (8) The development of isocyanate/tannin copolymers as difficult-to-bond hardwood adhesives and for plywood and other applications [25,26]. (9) The development of very low formaldehyde tannin adhesives for particle boards and other wood panels. (10) The development of the use of hardeners other than formaldehyde for thermosetting tannin adhesives [2,27,28]. (11) The discovery and development of self-condensation of tannin for adhesives [29,30,31,32,33,34,35,36].

All industrialized technologies today are based on paraformaldehyde or hexamethylene tetramine (hexamine) [36]. The latter is much more user and environmentally friendly.

As regards wood adhesives, a number of experimental improvements have been studied, dictated by the new environment in which wood adhesives must operate. First of all, the relative scarcity of tannins produced in the world, compared to the tonnage of synthetic adhesives used in the panel industry, has led to a great deal of research on the extension of the tannin resource in order to have larger tonnage. As the potential material for tannin extraction shows that millions of tons of this material can be extracted each year worldwide, some companies have started to build additional extraction plants. This movement is still relatively small, but it is ongoing. The second approach, to extend the tannin with another abundant and natural material, has led to the preparation of adhesives based on in situ copolymers of tannins and lignin [37] or copolymers of tannin and protein or soy flour [38], and the use of tannin–furfuryl alcohol adhesive formulations, furfuryl alcohol being also a bio-based material [39].

The second new constraint is the demand of most companies to eliminate formaldehyde emissions from tannin adhesive. This quest has taken two approaches: (1) total elimination of formaldehyde by substituting it with aldehydes, which are less or non-toxic, and non-volatile [28,40], such as glyoxal, glutaraldehyde, or vanillin, the latter giving a fully bio-based tannin adhesive, and even aldehydes generated by the action of sodium periodate on glucose, sucrose and even oligomeric carbohydrates, (2) the use of non-aldehyde hardeners such as trishydroxymethylnitromethane [41] and trishydroxymethylaminomethane [42] or even by combination with furfuryl alcohol, the latter functioning both as a hardener and a contributor to a tannin/furan copolymer [39,43]. (3) The use of hexamine with the formation of –CH_2_–NH–CH_2_– bridges between the tannin molecules, where the secondary amine is capable of absorbing any emission of formaldehyde from the heating of the wood itself or any other emission of formaldehyde to produce truly zero-formaldehyde emission panels [36,44,45,46]. (4) Lastly, the hardening of the tannins by autocondensation without the addition of a hardener, autocondensation catalysed by the wood substrate itself in the case of fast-acting procyanidin tannins, such as pine bark tannins, and for slower tannins by addition of silica or silicate or other accelerators [10,29,30,31,32,33,34,35] allowing the preparation of wood particleboard of indoor quality.

### 2.3. Pharmaceutical and Medical Applications

Tannins are known bactericides because they react with proteins irreversibly, thus complexing within bacterial membranes, neutralizing their activity. As a consequence, tannin-based pharmaceuticals to cure intestine infections have long-time been marketed. They have effective anticaries properties. Tannins have also many applications for other pharmaceutical/medical uses but all these are targeted for future use rather than the present.

Several experimental studies on the pharmaceutical use of tannins have been published with antitumor and anti-oncogenic activities particularly well documented [47,48,49,50,51,52]. Their antiviral effectiveness is also well documented by in vitro screening for a variety of 12 different hydrolysable and condensed tannins [51]. The tannin’s minimal inhibitory concentration (MIC) needed for reducing by 50% the cytopathogenicity induced by a number of viruses was used as an evaluation of their effectiveness. The lower MIC values yielded the best antiviral behavior. The different tannin’s minimum cytotoxic concentration (MCC) needed to detect microscopic alteration of normal cell morphology was also determined. Less toxic is the tannin tested in the patient’s cells, thus the higher is its MCC value, the more acceptable it is as an antiviral compound. The ideal antiviral compound is then the one presenting a combination of the lowest MIC and the highest MCC. The effectiveness of different tannins due to their polyphenolic nature can be very high against a number of different viruses. This is due to their irreversible reaction and combination with the viruses capsid proteins. It is the same reaction used in leather tannins and in their association with carbohydrates.

Thus, a number of commercially available tannins have been tested, namely mimosa bark tannin extract and its derivatives, chestnut tannin extract, tara tannin, quebracho wood tannin extract both sulphited and natural, pecan nut tannin extract, pine bark tannin extract, sumach tannin extract, and spruce tannin extract [51]. The viruses against which all these have been tested are highly varied, such as HIV-1 and HIV2, Herpes simplex 1 and 2, Vaccinia virus, vesicular stomatitis virus, Coxsackie virus B4, respiratory syncytial virus, Influenza A H1-N1, Influenza A H3-N2, Influenza B, Human Corona virus, Reovirus-1, Feline Corona virus, Sindbis virus, para-influenza 3 virus, and Punta Toro virus [52].

The inhibitory effects of these tannins have also been tested on proliferation of murine leukemia cells, murine mammary carcinoma cells, and human T-cells [51].

Acutissimin A is a bound flavonoid with an ellagi-tannin. It is formed by the interaction of a wine flavonoid with the vescalagin generated by the barrel’s oak tannin [53,54]. Acutissimin A has been found to present an effectiveness 250 times higher to stop tumors growth than the drug Etoposide.

While many studies have been conducted on a variety of tannins derived from a wide variety of plants as an anticancer treatment, some studies on the possibility of using tannin for other medical applications have also been highlighted. Condensed tannins are traditionally used for the treatment of intestinal problems [55,56]. This is due to their complexation ability with other molecules and their antioxidant behavior. The extract of *Stryphnodendron rotundifolium* and of other tannins has proven their effectiveness against ulcers by functioning as a protective coating of the gastrointestinal tract [57,58,59]. Other possible mechanisms of action of phenolic plant extracts as herbal medicines against ulcers and gastritis have also been described [57,58,59,60].

### 2.4. Wine, Beer, and Fruit Juices Additives and Antioxidants

Wine, beer, and fruit juices naturally contain tannins [61]. It is actually their presence that accounts for their characteristic taste. In short, the level of tannin in any of these products must be within a definite interval/concentration range for the beverage to be organoleptically pleasing. Too low an amount of tannin and the beverage will be insipid and with no taste. Too high the proportion of tannins in the beverage and it is too unpleasant, too “tanning” for the consumer’s mouth. Many wines, some beers, and several fruit juices however contain too low a concentration of tannin and thus may need to be “doctored”. Initially, addition of tannin or tannin-rich wood chips directly to wine to enhance its taste and give the impression of a wine of greater age was strictly forbidden in most European countries. With the determined and successful push for wine markets by southern hemisphere producers where limitations on adding oak particles to the wine to accelerate its aging was not forbidden, producers of more established countries tried to defend their market in a different way. Some producers the wines of which were particularly low in tannin content started to add tannin directly to some of their wines. Although addition of additives for aging was legally forbidden the perfectly legal gap existed permitting the use of tannins to precipitate proteic matter in the wine to render it “clearer”. All what was needed then was to add more tannins than what would be required to render the wine “clear”. This was kept fairly confidential, to not incur potential problems. The situation changed dramatically once the so-called “French paradox” came to be known. Namely, notwithstanding that traditional French diet is very rich in fats that should lead to grave cardiovascular diseases this is not the case, and this type of disease was far less frequent in France. This was ascribed to the regular use of red wine which decreased to extremely low level the risks and the occurrence of cardiovascular diseases. Now not only is tannin added to the wine, but it is considered particularly beneficial to do it. It must be pointed out that it is particularly purified tannin, from which carbohydrates and other components have been eliminated. It is the antioxidant property of the polyphenolic groups of the tannin which gives to it the powerful anticardiovascular effect and positive properties. Both addition of tannin as well as addition of tannin-rich oak wood chips to wine is now completely legal in Europe.

Equally, some beers need addition of purified hydrolysable tannins to acquire the proper taste. The same is valid for fruit juices.

As a consequence of the public awareness generated by the “French paradox” diet complements rich in flavonoid and hydrolysable tannins are on sale “over the counter” in North America.

This antioxidant effect of tannin is now starting to be exploited for foodstuff. The antioxidant effect of tannins is due to the characteristic of any phenol to stabilize free radicals and to inhibit further damage these may otherwise cause. Several factors influence radical control, these being [58] (1) the tannin colloidal state in water, (2) the special conformation around the interflavonoid bond, (3) how easy it is to open the pyran C-ring heterocycle, (4) the relative A- and B-ring hydroxygroups proportions, and (5) how easy is to solvate the tannin. These are the parameters determining the capacity of a tannin to act as an antioxidant.

A tannin’s antioxidant capability can be defined by measuring two different variables:

(1) How fast a tannin can form a radical, and uptake a radical by transfer from another radical species. Thus, the ease and ability of a tannin to capture free radicals from another species. The antioxidant ability is greater the easier and faster the radical capture.
(2) How quickly a tannin phenoxyl radical decays, thus how stable the radical is as a function of time. The slower the radical decay the lesser the radical degradation of the material to which tannin is added, hence the better the antioxidant properties of the tannin.

### 2.5. Fireproof and Insulating Foams

There are a number of different developments in rigid foam insulation. For imitating synthetic polyurethanes, by reaction with isocyanates, two approaches have been tried. First, foams have been developed based on the reaction of a modified tannin either by benzoylation or by oxypropylation to make it more susceptible as polyol to the reaction with polymeric isocyanates [62,63,64,65]. This first approach follows the same approach that was made with another natural polyphenol, namely lignin [66]. This is a traditional approach where tannin only functions as a polyol. The second approach of this type uses a very different strategy based on the reaction of a tannin with an aldehyde and the subsequent reaction of the methylol (–CH_2_OH) groups so formed with the isocyanate. This approach is now used for both tannin wood adhesives [25,67] and other formaldehyde adhesives such as urea-formaldehyde (UF), melamine-urea-formaldehyde (MUF) and phenol-formaldehyde (PF) [25,26]. Incidentally it is the only system that allows the formation of urethanes in an aqueous medium.

**Figure 5:**
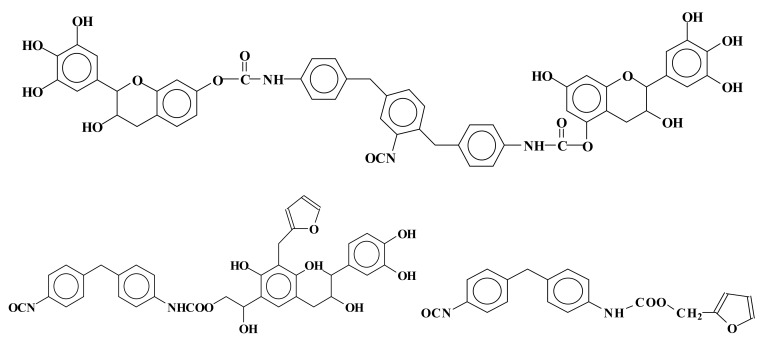
Examples of mixed species obtained by the reaction of tannin–furfuryl alcohol–glyoxal mixes with polymeric diphenyl methane isocyanate (PMDI).

Thus, according to this second approach, mixed rigid foams of phenolic–polyurethane type have been developed by reacting natural tannin/furanic mixtures with polymeric isocyanate in the proportions suitable for continuous polyurethane foam lines [68]. Urethane linkages formed at both the alcoholic C3 and phenolic hydroxyls. Other species in the mix were involved in urethane linkages formation: (1) glyoxal after or before its reaction with the tannin, (2) the phenolsulfonic acid catalyst, and (3) furfural. Furfural instead preferred to form methylene bridges with the flavonoids A ring than to form urethane linkages by reacting with the isocyanate. Thus, methylene and urethane bridges were formed between all the main materials in the mix. Thus, a number of mixed species bound by both types of linkages were formed. Species formed by mixed co-reaction of two, three, and four different reagents in the mix were identified. Examples of mixed species are shown in Figure 5:

Testing on a continuous production line has yielded positive results. This system was tried in plant trials in Switzerland on a continuous production line for polyurethane foam mats where addition of a small amount of isocyanate was necessary as without it the equipment of the factory could not function [68].

**Figure 6:**
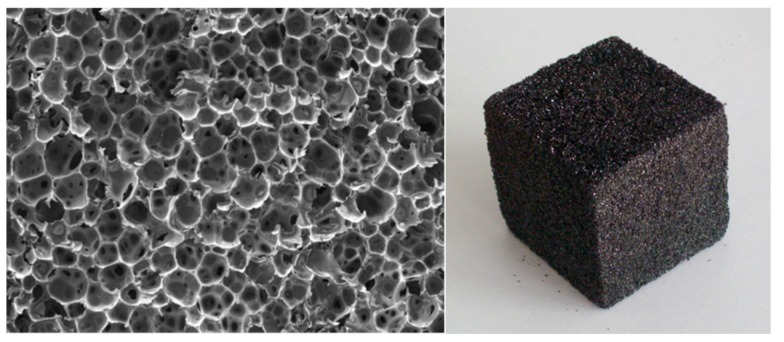
Scanning electron microscope image of the structure of a first-generation tannin–furanic foam (**left**) and macro-appearance of the same (**right**).

Chemically self-expanding rigid foam formulations based on tannin extracts have been developed since 1994 [69]. These foams, composed of 95% natural materials, have mechanical and physical properties comparable to synthetic PF foams. Originally, the fluid phase before foaming is composed of a tannin, with formaldehyde as a hardener, both mixed with furfuryl alcohol used as a exothermic reaction agent by its self-polymerization reaction and its reaction with the tannin [69] under acid conditions. Expansion to a foam of the fluid phase is caused by a low-boiling physical blowing agent, while simultaneous cross-linking of the resinous mixture provides dimensional stabilization at the desired low density [69]. These foams have been tested and are totally fire resistant, this being their major interest. Their appearance is shown in Figure 6.

During the last ten years a great deal of research has been carried out to improve these foams, by formulating them without formaldehyde [70,71], without aldehydes, without an organic solvent as blowing agent [72], without furfuryl alcohol, in an alkaline medium [73] and not only acid, with open cells and closed cells, by copolymerizing them with synthetic resins such as phenolic resins [74] and isocyanates [75], by copolymerizing them with proteins [76], by introducing variations into their systems of preparation [77,78], by improving their resistance to water by grafting on a small proportion of the tannin of long hydrorepellent chains [79], and also foaming them by heat alone without self-blowing, or even simply foamed by mechanical agitation for use as projected foams [80,81]. In addition to rigid foams, flexible foams have been developed [82,83,84]. Foams with the most reactive tannins of the procyanidin type, such as pine tannin, were also developed [85,86] and the carbonization line of research of these foams has also been continued and improved [87]. The varieties of these foams are really considerable [88] and the literature on this subject is really quite large, too vast to be summarized in this article.

A number of applications have been developed for these foams. Among others, open-cell foams have been used for very good acoustic insulation [89], carbonized foams for a large number of different applications [87,90], and also more recently for bone repair by osteogenesis with stem cells [91]. Taking the latter case as an example, for this application, as the treatment options are limited, bone tissue engineering opens the possibility of growing an unlimited amount of tissue products with increased therapeutic potential for the management of clinical cases characterized by severe bone loss. Bone engineering relies on the use of conformal biomaterial scaffolds, osteocompetent cells, and biologically active agents. Among other things, porous tannin spray dried powder (PTSDP) has been approved for human use. Thus the powder derived by grinding tannin–furanic foams has been used as a low-cost reconstruction material, due to its biocompatibility and potential ability to be replaced by bone in vivo. In this study, macro-PTSDP scaffolds with defined geometry, porosity, and mechanical properties were made by combining casting technology and pore leaching by mixing PTSDP and hydroxyapatite (Ca_10_ (PO_4_) _6_ (OH)_2_. This has shown that the scaffolds developed in this work support the attachment, long-term viability, and osteogenic differentiation of mesenchymal progenitors derived from human induced pluripotent stem cells. The combination of some macroporous PTSDP scaffolds with patient-specific osteocompetent cells offers therefore new opportunities to develop bone grafts with increased clinical potential for complex skeletal reconstructions.

The variety of uses of these foams is potentially such that many other applications can be envisaged, not only envisaged at present but to be developed in the future.

### 2.6. Calcite Depressant in Ore Flotation and Other Mining Applications

Unmodified mimosa bark and quebracho wood tannin extracts are used as depressants for unwanted calcite for the recovery of fluorspar in South Africa by a single mining firm. Consumption for such an application is relatively low at about 100 tons of tannin extract per month, and the extract is applied at the rate of 1 kg tannin per ton of low grade (20%–25% CaF_2_) fluorspar ore [92].

Equally low is the consumption of tannin extracts for the separation of germanium from copper in the big open air copper mine in Chile. Due to the low percentage of the very expensive and rare gemanium in the copper ore, this is treated with flavonoid tannins, the B-rings of which complex preferentially with the germanium, allowing its separation. The tannin–germanium complex is then burned and the germanium recovered.

### 2.7. Flocculants and Precipitation of Polluting Materials by Complexation of Heavy Metals

Scavenger behavior of natural flavonoids for several kinds of metal ions is well-known in literature [93,94,95,96]. On the other hand, nowadays, the problem of purification of wastewater by heavy metal ions is more and more considered because there is a growing sensitivity around their environmental pollution [97,98].

The idea of using a tannin in powder form, or as a rigid foams to purify industrial wastewater, connects the natural ability of polyphenols to entrap metal ions with the easy removal system of such an innovative product [99,100,101,102,103].

A reliable proportionality has been found between initial concentration and percentage of metal ion adsorbed. The most important aspect to consider is that tannins are able to adsorb copper (12.5%) and lead (20.1%) ions in their structures [102]. Moreover, analysis results show that the adsorbing materials are not exhausted and it is possible to preview a scavenger activity even for more polluted water solution. More recently, *Pinus pinaster* tannin extracts have been used for the complexation and precipitation of antimony Sb ions [104].

**Figure 7:**
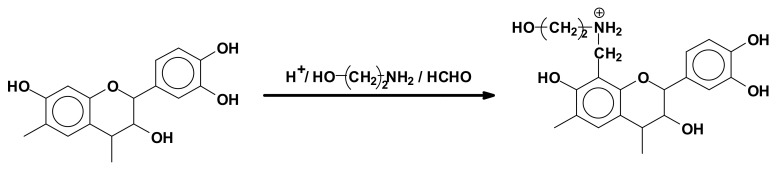
Reaction of ethanolamine and formaldehyde with a flavonoid tannin unit to form anpho-tannins used as industrial flocculants.

Tannin extracts have also been used as flocculants for clay suspension in water treatment. Strongly acid conditions are used to link ethanolamine with mimosa tannins which are water soluble (Figure 7). These anpho-tannins have been used in South Africa for over 15 years as flocculants for clay suspensions in municipal water treatment. In this process no residual salts or ions remain in the treated water, the anpho-tannins combining with the suspended clay and coprecipitating [92].

More recently, research has continued on the use of tannins as coagulants of particles in polluted waters to eliminate color even in wastewater by modifying the tannin by the Mannich reaction [105].

### 2.8. Inhibitors of Corrosion of Metals

Chemical and electrochemical acid corrosion of iron, steel, and alloy are common place. This causes great losses because the metal parts affected have to be replaced. Thus, anticorrosion coatings are a practical way of prevention. Metal surface preparation is essential when using such methods of corrosion prevention. An anticorrosive primer added before the main paint will markedly improve the protection of the substrate imparted by the paint finish. Many researchers have studied the anticorrosive properties of tannins. Tannin-based primers for the protection of metal surfaces and as anti-corrosion agents were already available on the market in the 1950s.

An anticorrosion primer formulation for steel has been published by Matamala et al. [106], this being primary layers of pine bark tannins that could prolong the paint life by more than 50% if applied before the main paint. In addition, tannins can be used as anticorrosives together with specific solvents and added to other materials such as epoxies, zinc oxide, or copper. This improves the quality of tannins to the same level of traditional anticorrosive primers. Such primers are applied by brushing or other suitable methods. Both hydrolyzable and condensed tannins can function as anticorrosive metal primers, as they both can potentially oxidize phenolic groups (antioxidant) and complex the metal substrate by forming of orthodiphenol metal complexes.

**Figure 8:**
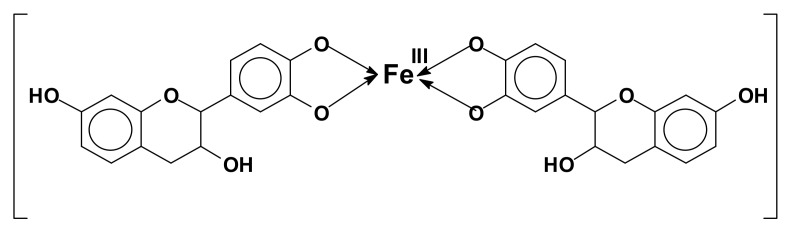
Example of orthodiphenol iron complex of a flavonoid tannin.

The reason for the coupling of tannins with metals is, for example, the formation of Fe complexes, used to prepare intensely black/violet inks by formation of ferric tannates, and also as anticorrosion varnishes. These coordination complexes are due, for example, to the orthodiphenol hydroxyl groups on the B rings of a flavonoid tannin [107] (Figure 8).

Ship hulls are traditionally coated with metal-containing anti-fouling paints. These provide protection by releasing a toxic compound. Tannins and copper can be used as antifouling for ship parts in seawater and reduce foulings. In addition, such formulations were able to reduce copper consumption by 40-fold compared to copper-based paints. It is this same type of complexation that has given the ferrous tannate-based inks still used in school in the first half of the 20th century and the use of which had its peak in the middle ages (cf. inks).

### 2.9. Core Binders for Foundry Sands

Foundry cores of high strength have been produced using both hot and cold-set tannin–formaldehyde adhesives to bind mold sands [92]. The mimosa or quebracho tannin–resol, which may be prepared and stored in solid form, reacts with more tannin extract (when mixed in the proportion 1:3 under heating and stoving at a temperature up to 170 °C). In cold-setting applications, setting of the resol with resorcinol may be acid catalyzed with ammonium chloride and p-toluene sulfonic acid. Setting of the cores from the cold-setting mix can be accelerated at any time by stoving.

### 2.10. Mud Stabilizers and Drilling Fluids

Drilling fluids play important roles in the drilling, such as in geothermal drilling programs, and serves several purposes in the circulation system. Among those are (1) cooling and lubricating the drilling strings while it is circulating, (2) transporting the cuttings that are created at the bottom of the hole and release them at the surface, (3) controlling the formation pressure while maintaining the wellbore stability, (4) sealing the permeable formations across the wellbore, and (5) assisting the logging operations. In drilling operations, the basic properties of drilling fluids are usually defined by the well program and monitored including rheology, density, fluid loss, solid content, and chemical properties. To avoid any drilling problems such as a blowout, stuck pipe, and other problems, engineers are always searching for a good performance of a drilling fluid system that is fit for the purpose. Dealing with standard temperature and pressure conditions, the properties of drilling fluids are much easier to handle. While in hostile drilling conditions like high temperature environments, the selection of a drilling fluid is more crucial, alongside the lithology that is one of the factors to be considered. The high temperature environment is often said to be at a temperature more than 150 °C. In geothermal drilling, the down hole temperature can reach up to 250–350 °C at the depth of 1000 to 2000 m. At this temperature, the selection of a drilling fluid becomes more stringent due to the needs of thermal stability of the drilling fluid system as well as materials used in the system. High temperatures can cause chemical alterations of various drilling fluid components, thus affecting the filtration and viscosity as well as increasing the tendency for gelation. In addition to this, clay activities greatly affect the system, with high tendencies to flocculate and gel.

Sulfited quebracho tannin extract has been traditionally used as a mud-thinning agent in shallow drilling [92]. However, stricter criteria of stability and anticorrosive action at elevated temperatures have been introduced in recent years. Thus, an effective conditioning agent for bentonite mud has been developed by incorporation of chrome salts into mimosa tannin extract [92]. This product is effective to a depth of 2000 m under normal drilling conditions and in absence of high salinity.

Tannin–lignosulfonate drilling additives that have been coreacted have also been prepared [108]. Calcium lignosulfonate (CaLS) is dissolved in an aqueous solution of hydrochloric acid for decalcification. The amount of CaLS added to the solution depends on the weight ratio between CaLS and the desired tannin. The mixture is frequently stirred for 10 min and sodium hydroxide is then added to adjust the pH of the solution to between 3 and 4. Then, the calcium sulfate is removed from the solution by filtration to obtain lignosulfonic acid. On a hot plate at a temperature of 105 °C, formaldehyde is added to the solution to initiate crosslinking. The tannin is then added according to the desired weight, and the solution is stirred continuously for 2 h. The ferrous sulfate heptahydrate is chelated with the solution for 30 min at the same temperature. Finally, the tannin–lignosulfonate solution is dried in a vacuum oven at a temperature of 60 °C for 48 h. The evaluation of the decomposition rate by thermogravimetric analysis (TGA) provided proof of the thermal stability of the tannin–lignosulfonate. The thermal stability of tannin–lignosulfonate has been identified at 294 °C and more than 60% of the weight remains at this temperature. It was found that the gel strength at 10 s and 10 min was reduced with the addition of deflocculant in each sample. [108].

### 2.11. Teflon/Metal Adhesives Resistant to High Temperatures and High Temperature Resistant Surface Finishes for Metals

**Figure 9:**
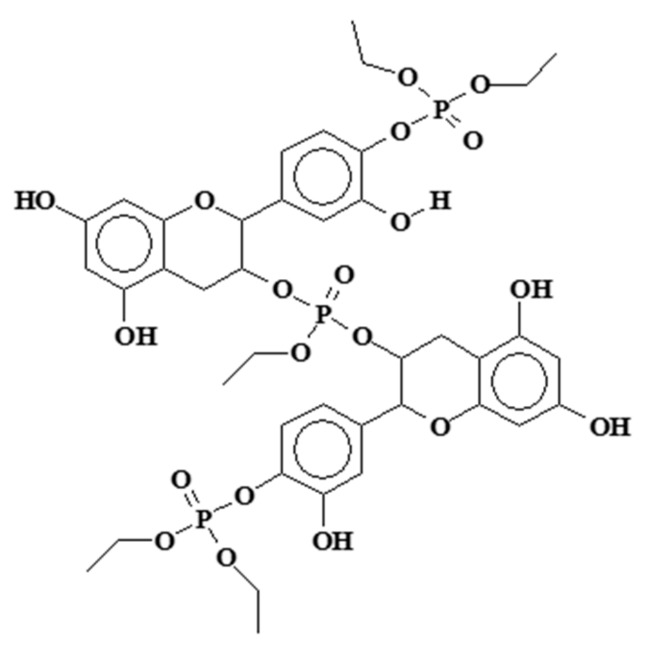
Example of cross-linking product of a flavonoid tannin by reaction with triethyl phosphate [109].

The condensation and crosslinking reaction of mimosa tannin extract and a flavonoid monomer model with triethyl phosphate (PET) has been studied [109] (Figure 7). The reaction was shown by multiple instrumental analytical techniques to occur at the C3 of the flavonoid heterocycle and the flavonoid B-ring’s C4 and C5 aromatic carbons, but not on the flavonoid A-ring. The relative proportions of reaction on these two sites differed for tannin and monomer model compounds. The reactions is temperature-dependent. The reaction, which takes place at temperatures around 180 °C, leads to hard solid finishes and films particularly suitable for attachment to aluminum and stainless steel and presents high thermal stability. Their potential use for which they were initially developed is in particular for the attachment of teflon coatings on non-stick steel or aluminum frying pans [109,110]. Subsequent testing with either of these tannin-based adhesives or other natural polyphenols [111] has demonstrated resistance to temperatures in excess of 400 °C for certain periods of time. Ammonia and high temperatures favor these reactions. The first application tests performed at high temperatures showed good performance as a metal coating. The type of polymers that are formed are as shown in Figure 9.

### 2.12. Cut Flowers and Hydroponic Horticulture Foams

High water absorption and retention, good air access, lower density for easy flower insertion, and particularly optimal pH to ensure durability are the required properties for synthetic phenol–furan synthetic foams for floral, hydroponic, and horticultural uses [112,113,114,115].

**Figure 10:**
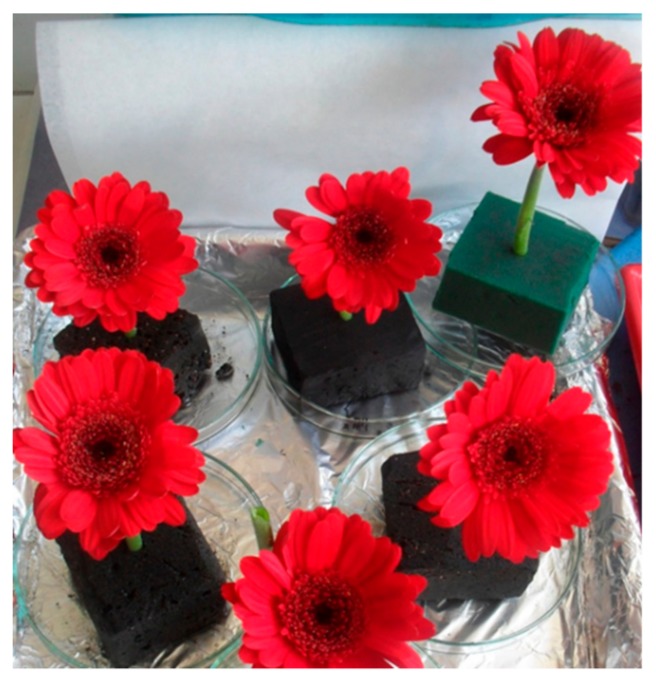
Appearance of cut flowers (Transvaal daisies, *Gerbera* spp) on a tannin–furanic foam (black) compared to a synthetic phenol–formaldehyde/furanic foam (green).

New quebracho tannin–furfuryl alcohol foams without formaldehyde have also been developed for the same applications [116] (Figure 8). Compounds to neutralize the inherent residual acidity of the catalyst used as a wetting agent are always included. Densities were in the 0.048 to 0.066 g/cm^3^ range and compression strengths in the 0.07 to 0.09 MPa range. They present open pores with average cell sizes in the 125 to 250 μm range, peak water absorption up to 98% by volume, and a residual pH of 5. They do not present any phytotoxicity for preserving freshly cut flowers and are good supports for horticultural hydroponic cultures [116] (Figure 10).

Vermiculite and dolomite are added as well. The foams’ pH determines their performance for rendering water and air available to the plants. The amount and structure of the anionic surfactant used also determines the water and air availability of the foam [116]. The inclusion of antifungal compounds and nutrients in the foam composition is also beneficial. Tannin foams based on renewable natural materials not only are environment-friendly but showed performance comparable to or better than the commercial synthetic phenolic floral foam used as a reference [116].

### 2.13. Corrugated Cardboard Adhesives

The adhesives developed for the manufacture of damp ply resistant corrugated cardboard are based on the addition of spray-dried wattle extract, urea-formaldehyde resin, and formaldehyde to a typical Stein–Hall starch formula of 18 to 22% starch content [16,117]. The wattle tannin–urea–formaldehyde copolymer formed in situ, and any free formaldehyde left in the glue line was absorbed by the wattle tannin extract. The wattle extract powder should be added at level of 4 to 5% of the total starch content of the mix (i.e., carrier plus slurry). Successful results can be achieved in the range of 2 to 12% of the total starch content, but 4% is the recommended starting level. The final level is determined by the degree of water hardness and desired bond quality. This wattle extract–UF fortifier system is highly flexible and can be adopted to damp-proof a multitude of basic starch formulations. This system has been in operation for some decades in countries where tannin is easily obtainable.

### 2.14. Non Isocyanate Polyurethanes (NIPU)

A high yield of urethane linkages was obtained by reacting hydroxyl-rich chestnut hydrolysable tannin with dimethyl carbonate followed by hexamethylenediamine [118]. Polyurethanes prepared in this way are of interest as toxic isocyanates and are not used as reagents, and their main constituent is instead a renewable material.

**Figure 11:**
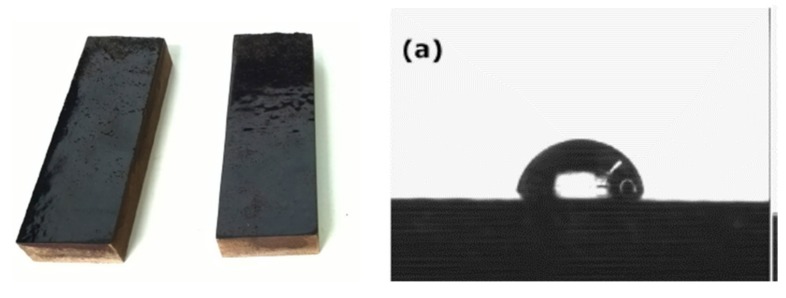
Non-isocyanate polyurethane-based (NIPU) wood surface coatings based on condensed tannins (**left**) and sessile water drop contact angle on the NIPU coating surface (**right**).

The same approach has been applied to condensed tannins. Several condensed tannin extracts were used for this purpose, namely: the bark tannins of maritime pine (*Pinus pinaster*), of mimosa (*Acacia mearnsii*), and radiata pine (*Pinus radiata*), and the wood tannins of quebracho (*Schinopsis lorentzii and balansae*). These were initially reacted with dimethyl carbonate followed by hexamethylenediamine forming urethane bonds [119]. Wood surface finishes based on these poly(hydroxyl)urethanes yielded encouraging performances (Figure 11) with high sessile drop contact angles on the treated wood surface (Figure 9).

**Figure 12:**
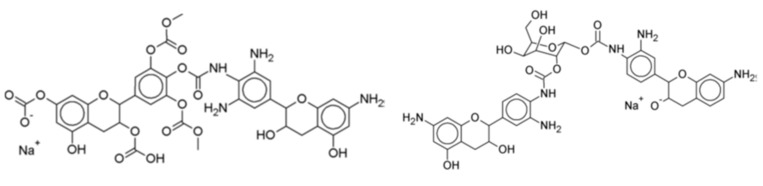
Examples of compounds presenting urethane linkages formed by the reaction of a pre-aminated flavonoid tannin with a flavonoid tannin pre-reacted with dimethyl carbonate.

Furthermore, to increase considerably the proportion of bio-sourced material in these isocyanate-free polyurethanes, pre-aminated mimosa tannin extract (as a substitute of the synthetic hexamethylene diamine) was reacted with a mimosa tannin extract pre-reacted with dimethyl carbonate [120] (Figure 12). This reaction proceeded easily at room temperature. Cured above 100 °C, the polyurethanes formed yielded a hard film [120]. In addition, not only the polyphenolic fraction of the tannin extract, but also the carbohydrates oligomers in it generated isocyanate-free polyurethane linkages with the aminated tannin [118,120], indicating that non-isocyanate polyurethanes can be synthesized just from carbohydrates. The compounds formed were identified by several instrumental analytical techniques. Species as shown in Figure 12 were identified.

**Figure 13:**
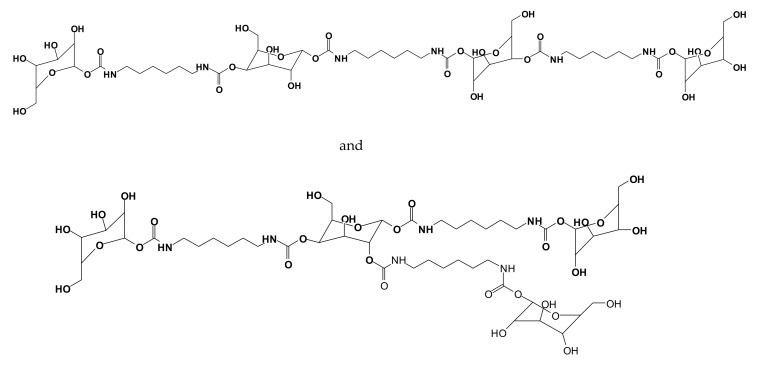
Examples of linear and branched glucose-based non-isocyanate polyurethane oligomers [121].

The reactivity of the carbohydrates present either in the condensed tannins or in the hydrolysable tannin and the isolation of polyurethanes without isocyanates (NIPU) formed with the carbohydrates or carbohydrate–tannin mixtures led to the evolution of simple monosaccharides and disaccharides as a base of NIPU [121]. Thus, predominantly bio-sourced isocyanate-free polyurethanes (NIPUs) were developed from glucose and sucrose by reaction with dimethyl carbonate and hexamethylenediamine [121]. Oligomers obtained were detected by several spectrometry techniques, showing linear and branched structures such as those shown in Figure 13.

Glucose-based NIPUs cured at a noticeably lower temperature and were more easily spread than sucrose-based NIPUs. Wood and steel surface coatings were prepared using these NIPUs. The sessile drop test (contact angle, on wood) and the cutting grid test (on steel) of these NIPUs yielded very encouraging results [121]. Glucose NIPUs gave good results as a surface coating curing at 103 °C, whereas sucrose NIPUs performed well only at a much higher curing temperature. NIPU resins derived from saccharides have also been used as wood panel adhesives. The glucose-based NIPU gave very encouraging results for this application [121] while the sucrose-based NIPU gave very encouraging result for the preparation of polyurethane foams.

### 2.15. Isocyanate-Based Polyurethanes

**Figure 14:**
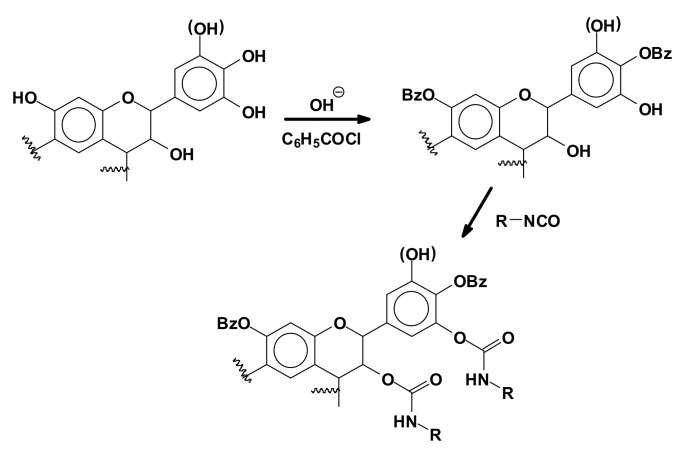
Reaction of a flavonoid tannin modified by benzoylation with an isocyanate.

To obtain synthetic polyurethanes using tannin as a polyol, thus by reaction with isocyanates, a first attempt was made quite early-on by direct reaction of an isocyanate with the hydroxyl groups of a condensed tannin [62]. A much more recent second approach was based on the reaction of a tannin modified either by benzoylation (Figure 14) or by oxypropylation to introduce hydroxygroups on the tannin that are more easily approachable for reaction and more likely to react as a polyol with polymeric isocyanates [63,64,65].

This first approach follows the same approach that was made with another natural polyphenol, lignin [66]. This is a traditional approach where the tannin only functions as a polyol. Nevertheless, while tannin-based polyurethanes are indeed obtained by this route, other natural polyols do exist that are much more adapted to the formation of polyurethanes by reaction with isocyanates, and moreover, without needing to add an additional reaction such as oxypropylation or others.

### 2.16. Hard Thermoset Plastics and Resins for Angle Grinder Disks and for Automotive Brake Pads

New 100% bio-sourced, tannin/furan hard thermosetting plastics were prepared by copolymerization and characterized [122]. This new material is synthesized by the reaction of tannin and furfuryl alcohol, two inexpensive vegetable chemicals. The co-polymerization processes of both were investigated by ^13^C NMR and matrix assisted laser desorption ionization time of flight (MALDI-TOF) mass spectrometry. The tannin/furan thermosetting resin with 100% renewable organic material has a glass transition temperature of up to 211 °C and a 95% weight loss temperature of 244 °C and 240 °C in a nitrogen atmosphere and in the air, respectively [122]. The yield of carbonization achieved is 52%. In addition, this new thermoset material has excellent mechanical properties: the Brinell hardness is superior to commercial acrylic plastics, polyvinyl chloride, and slightly lower than solid polystyrene. Compression strength was found to be as high as 194.4 MPa, thus higher than that of filled phenolic resins, and much higher than that of solid polystyrene and acetal resins [122].

A tannin–furfuryl alcohol thermoset resin [122,123] has also been used as a resin matrix for solid grinding wheels [123], these being also tested and their performance characterized and showing good abrasion comparable to commercial grinding wheels. This resin uses only bio-sourced raw materials. Moreover, its process of preparation is simple, and thus easily transferred under industrial conditions. This resin has been used to bond different mineral and organic abrasive powders to prepare the grinding wheels.

**Figure 15:**
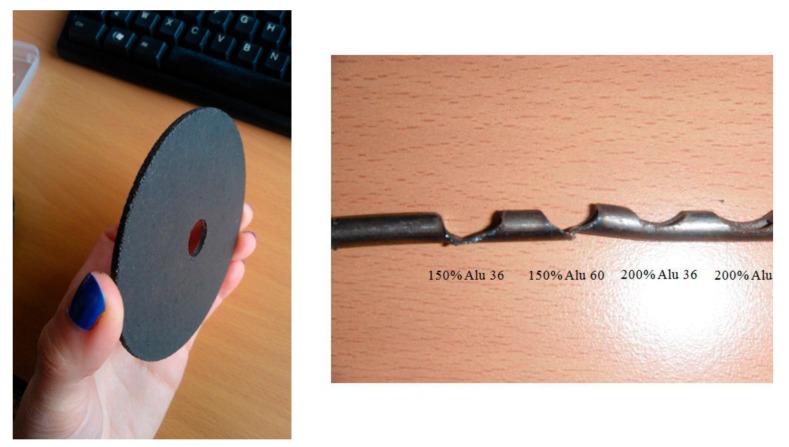
Example of an angle grinder disk using a tannin–furfuryl alcohol resin as a matrix, and example of cuts in a steel bar with it when using different abrasives.

The same bio-sourced tannin–furanic thermosetting resin has been used as a resin matrix for angle grinder disks, presenting excellent abrasion and cutting properties [123] (Figure 15). A fiberglass mesh support was used for the abrasive powder and the green resin matrix yielded a rather easy manufacturing process. Aluminum dioxide was the abrasive used. The mechanical strength of these grinder disks was comparable to their commercial equivalents using synthetic phenol–formaldehyde resins. They stood up well at 11,000 rpm to the high stresses induced by cutting or grinding steel [123]. Steel bar cutting times with these green experimental disks compared well to those of commercial synthetic resin-bonded disks.

Automotive brake pads bonded with a bio-sourced thermoset tannin–furanic matrix were developed and tested [124] using similar matrix resins. Their preparation is also quite easy. The braking and characteristics of such green brake pads tested under real-world large scale test conditions were excellent as well as their wear resistance [124,125]. Their performance compared well with that of synthetic phenolic resin-bonded commercial automotive brake pads. They stood up well to heavy braking, and strong stresses such as emergency braking at 50 km/h (31 mph) to a complete stop. The braking distances were comparable and sometime shorter than commercial brake pads.

### 2.17. Epoxy and Epoxy–Acrylic Resins

Hydrolyzed and condensed tannins have also been used to synthesize monomer epoxies. Thus, either epichlorhydrin was reacted with catechin to yield an epoxidized catechin monomer or alternatively by alkylating it with unsaturated halogenated compounds and then oxidizing [126]. Analysis of the reaction products shows the presence of a by-product of the benzodioxane group, which then decreases the average epoxy functionality [127].

**Figure 16:**
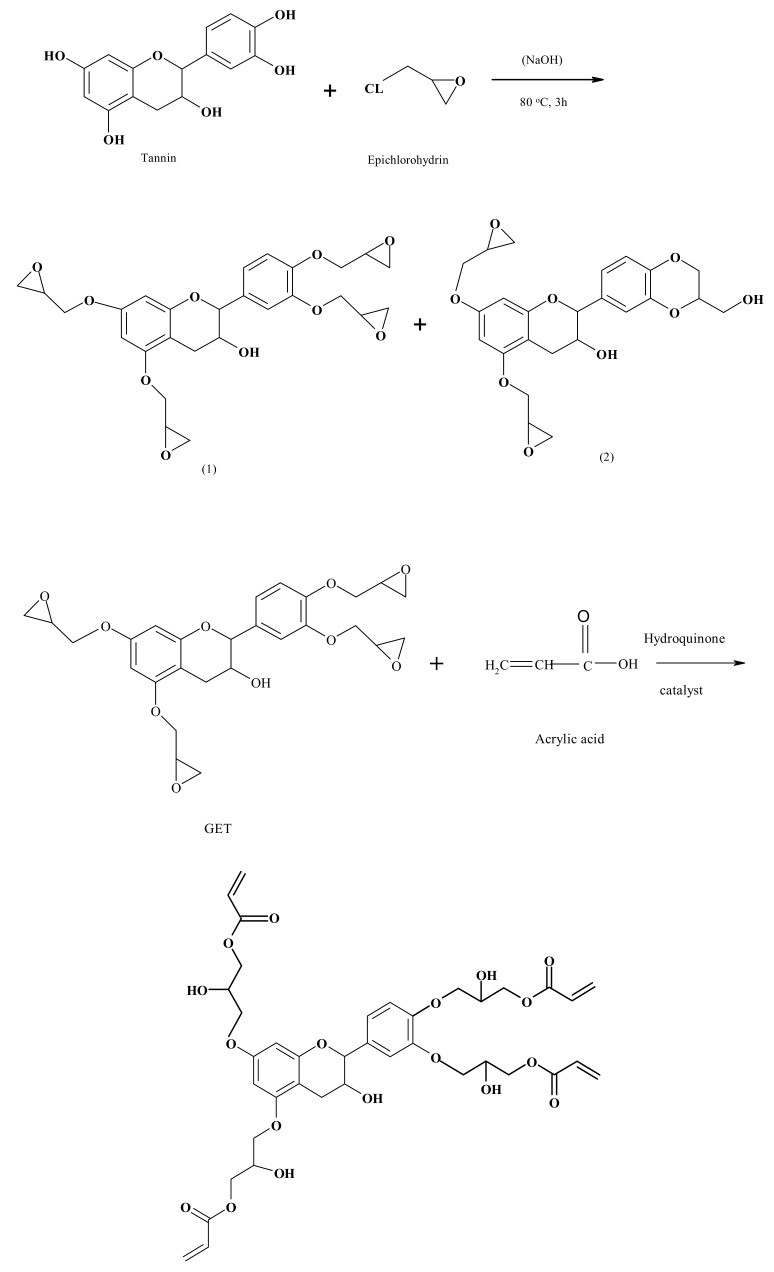
Scheme of the sequence of reactions to prepare the epoxy acrylate resin. The reaction proceeds in two steps: first, the epoxidization of the flavonoid tannin, and second, its reaction with acrylic acid catalyzed by hydroquinone [129].

Following the excellent seminal works cited above, more recent work has appeared on the preparation and characterization of condensed tannin epoxies [128,129]. One of these deals with the preparation an epoxy–acrylic tannin resin capable of quick curing without using a hardener [128,129] (Figure 16). Glycidyl ether tannin (GET) was the starting material for preparing the tannin epoxy acrylate resin by reacting it with acrylic acid with a catalyst and adding hydroquinone as well [129] (Figure 16). It was tested for shear resistance with very interesting results. Unlike the other studies that used monomers as models, these two studies were conducted with a commercial extract of mimosa tannin, thus in a real situation.

Tomita and Yanezawa first reported the epoxidation of gallic acid with epichlorohydrin [130]. The presence of an ammonium catalyst of phase transfer under anhydrous conditions promotes the addition of epichlorhydrin to the carboxylic acid function and to one phenol group at least. The epoxy equivalent weights (EEW) reported were between 137 and 160. This corresponds to a 1 to 4 epoxy functionality. The epoxidized compound is then cross-linked with a conventional crosslinking agent polyamine, or an anhydride is then used to cross-link the epoxy resin formed, thus by a traditional crosslinker [130]. Theoretical calculations, however, did show that Tomita and Yanezawa achieved an average epoxy functionality of 2, notwithstanding the use of four epichlorohydrins per phenol group.

The synthesis conditions of gallic tetra epoxy acid has been recently published [131]. Only resins with only two or three epoxy groups were reported before. This work established a relationship between different phenol monomer chemical structures and their reactivity with epichlorhydrin by studying the mechanism of *O*-glycidylation. Molecular understanding of this relationship is determinant for developing bio-sourced epoxy monomers derived from tannins [131].

Nouailhas et al. [127] have also recently proposed another synthetic route leading to epoxy gallic acid allylation prepolymers by reacting gallic acid with allyl bromide followed by double bond oxidation using m-chloro-perbenzoic acid. This method makes it possible to obtain epoxy prepolymers with epoxy functionality up to 3.

Green tea condensed tannins have also been proposed as suitable to synthesize bio-sourced aromatic epoxy oligomers, based on work on model molecules of tannins [132].

### 2.18. Tannin-Impregnated Fibreboards

**Figure 17:**
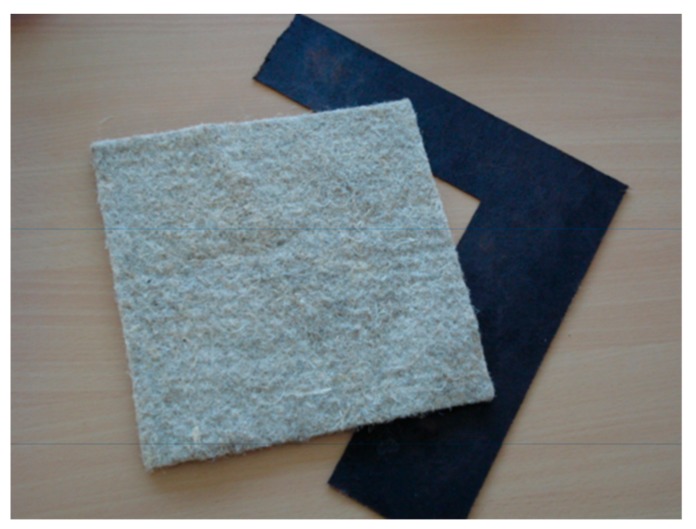
A nonwoven web of hemp fibers and the composite obtained by impregnation with a tannin–furanic resin.

Nonwoven flax and hemp fiber mats were impregnated with renewable bio-sourced resin matrices to yield both high and low density composites of good performance [133,134,135,136] (Figure 17). The types of bio-sourced matrices used were: (1) a 5% hexamethylenetetramine-hardened resin based on commercial mimosa tannin extract, and (2) a 50/50 combination by weight of glyoxalated organosolv lignin of low molecular weight and of mimosa tannin with hexamethylenetetramine. Modulus of Elasticity (MOE) in bending and tensile strength were tested for these composites to maximize tensile strength. Corona-treatment was also used to improve the tensile strength of such composites, and its most adequate duration determined. These composites were tested for different characteristics, such as surface hardness, water contact angles, influence of fiber morphology, and others, always yielding good results. The matrices based on mixed tannin and lignin gave composites with a thermoplastic behavior after just the first hotpressing. They were thus thermoformable as they could be shaped in their final form by a second hotpressing.

The hardening cycle, press temperature, press time, pressure, moisture content, and the number of fiber mats influenced the composite characteristics. Furthermore, first drying the resin-impregnated fiber mats for storing them and then rehydrating them before hotpressing allowed the mats to be maintained ready for use even after long storage times. This approach still yielded composites of 50% matrix resin/50% natural fibers presenting good tensile strength, water swelling, and Young’s modulus. The best results were achieved at a slow curing low temperature (130 °C, 35 min) at a 20% moisture content.

Acid catalyzed tannin/furfuryl alcohol resins in proportion 45/54% by weight were also tried for this application, yielding flax fiber lightweight composites with good performance.

### 2.19. Wood Surface Finishes

Tannin–furanic resin impregnated paper was applied as a surface finish to plywood with good results. Hexamethylenetetramine, paraformaldehyde, formurea, and a mix of the latter and paraformaldehyde were used as hardeners. These gave good results in both standard crosscut and water vapor tests. Chemical analysis has confirmed both the reaction of tannin with furfuryl alcohol and the autocondensation of the latter, as well as the co-reaction of both tannin and furanic aromatic rings with formaldehyde-yielding compounds such as hexamine. The oligomers formed were determined [137].

MUF impregnated paper surface coatings were compared with the tannin–furanic impregnated papers with regards to the surface quality of wood panels, with the latter giving better water vapor resistance and cross-cut testing. Abrasion resistance was influenced for both types of coatings by the impregnated papers moisture content. Color measurements indicated that a higher moisture content yielded a lighter color of mimosa tannin impregnated papers. Nonetheless, impregnated surfaces with tannin/furan resin impregnated paper are too dark for decorative applications, but may be useful for the manufacture of cement formwork panels [138].

By paper impregnation with a tannin–furanic resin hardened with a formurea concentrate, high pressure paper laminates were prepared. These were tested for crosscut, abrasion, and water vapor resistance. Ten-layer high pressure paper laminates of this type improved the dry shear strength of plywood to which they were applied as a surface coating as well reducing its water absorption. Paper laminates pressed for 600 s, at a 140 °C and 120 kg/cm^2^, yielded the best results. High pressure laminates impregnated with a tannin/furanic resin and creating thick materials compare favorably with the properties of synthetic high pressure thick laminates impregnated with synthetic phenol–formaldehyde resins as used in mechanical gears, etc. [139].

### 2.20. Flexible Plastic Films

**Figure 18:**
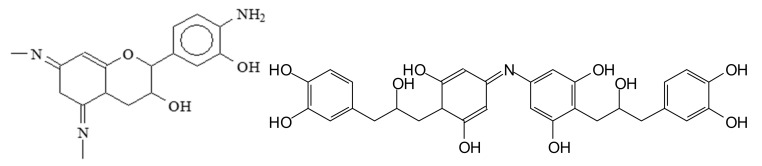

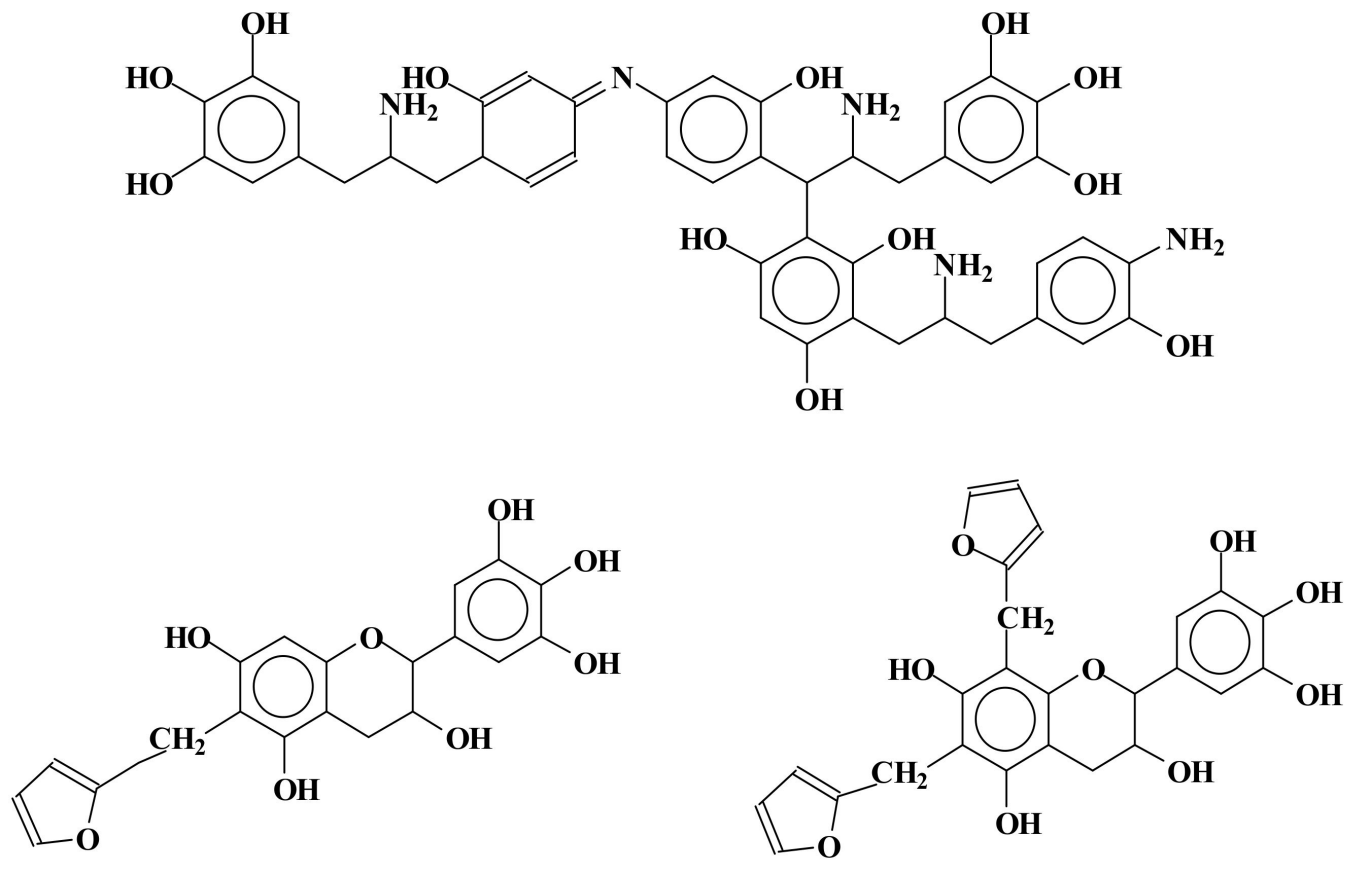
Examples of compounds prepared by amination of a flavonoid tannin, and simultaneous reaction of the tannin with furfuryl alcohol.

Flexible films and strongly adherent surface finishes have been prepared [140] by reacting partially aminated polyfluorocarbon tannins with furfuryl alcohol in the presence of plasticizers such as glycerol or polyethyleneimine. 13C NMR analysis shows a partial amination of the tannin under the conditions used and even the formation of –N= bridges between the flavonoids, although these have proved to be rare [102]. Thus structures as in Figure 18 have been observed [141].

MALDI-TOF analysis has shown the presence of oligomers produced by the reaction of furfuryl alcohol with flavonoid A-rings and the simultaneous self-condensation of furfuryl alcohol [140]. Thus, the methylene–furanic linear chains have also been shown to be linked to reactive flavonoid tannin sites. In addition, side condensation reactions of furfuryl alcohol led to the formation of methylene ether bridges between furanic rings, followed by rearrangement to methylene bridges with consequent release of formaldehyde. The latter reacted with both the flavonoid and furan reactive sites to give –CH_2_OH and –CH_2_+ groups and further methylene bridges [140].

### 2.21. Cement Superplasticizers

To avoid the retardation effect that common plasticizers have while fludifying cement, superplasticizers have been developed so that this retardation effect could be eliminated. They improve the facility of working cement, reduce the water needed, and improve the cement final strength. These materials transform cement pastes into flowing fluids. Commercial superplasticizers are synthetic resins such as sulfonated melamine–formaldehyde or naphthalene sulfonate–formaldehyde [52,53,54]. Modified lignosulphonates are also used for this category of materials [142,143,144]. Up to 30% water reduction are possible with slump sizes of 200 mm.

Their mechanism is based on their adsorption on the cement grain surface while maintaining the water orientation of their sulphonic groups. The water monolayer that is formed around the grain causes a dispersion of the grains contributing to the fluidification of the cement/concrete paste. Moreover, the surface tension of water is not much reduced and there is no significant retardation of cement setting or hardening [144].

The structures of polyflavonoid tannins have the ability to complex Fe^2+^/Fe^3+^ and aluminum ions in cement by the ortho-diphenol hydroxyls on their B-rings [2,10]. Sulphonation improves their solubility in water [2,10]. These characteristics make polyflavonoid tannins attractive as dispersants/superplasticizers for cement, composed mainly of Ca and Fe silicates and aluminates.

Several tannins, especially sulphited, have performed well as cement superplasticizers [145]. Extracts of sulphited mimosa, quebracho, and pine tannin all behave very well as cement superplasticizers, with mimosa and pine being those with slightly better behavior. A dosage of 0.25 to 0.5% by weight on the cement has a significant effect of fluidification.

Modified condensed tannin extracts behave as superplasticizers. Cement and concrete flow better and the onset of hardening is not retarded [145]. The total effect was due to a mix of different causes: (1) the silicate and aluminate cement components inducing an increase in the tannin molecular weight, (2) the improvement in tannin solubility due to the insertion of sulfonic groups leading first to the decrease in the tannin’s molecular mass and then to its stabilization, and (3) urea addition stabilizing the molecular mass by minimizing tannin colloidal interactions and hindering tannin rearrangement and autocondensation [145].

### 2.22. Ferric Tannate Inks

Ferric gallo-tannate ink is a black to purple ink made from metal salts, especially ferrous sulfate but sometimes copper sulfate, and various tannins of vegetable origin. Black ink, emblematic of the monastic scriptorium, was the most used ink in Europe between the twelfth and nineteenth centuries. This tannic ink or solubilized tannins is sometimes referred to as ferric ink, ferro-gallic, or metallogallic. Irreversible damage to paper due to this corrosive ink poses significant conservation problems. The particularity of this ink lies in its absence of pigment or dye; it is the action of metal salts (iron or copper sulfate) which, added to the tannic material (the gall nut), gives the black hue, most often dark violet before aging. Its defect is its corrosivity, for the paper as well as for the metal quill. To limit this inconvenience, its manufacture must be the subject of a good aging; maceration of the tannin for three months then, after mixing with the iron salt, a maturation of at least two months to one year guaranteeing its optimum. There is a plethora of different recipes. The three main constituents are:

(1) the gall nut (gallo-tannic acid) or various solubilized extracts of bark tannins (oak, etc.) which are possibly dried, (2) iron sulfate (ferrous sulfate). Sometimes copper sulfate can be used, but this, also corrosive, attacks the paper, and (3) a binder, such as gum arabic or wine lees (that is to say, wine whose liquid part has evaporated). This mixture is swelled in lukewarm water for one day. Salicylic acid or phenol may be added to prevent the development of microorganisms. It is hygroscopic (slows down drying) and keeps the ink particles in suspension and prevents them from precipitating (colloidal solution). In the fifteenth century, plum, apricot, or cherry resins were used to achieve this. The excess of ink so prepared can be dried. Full drying was done in pork bladders. The base ink (tannin) is then redissolved and a reagent is prepared, with iron sulfate dissolved in warm water, to which gum arabic is added. The tannin solution is boiled separately and gum arabic is also added. The mixture is allowed to cool and the two solutions are mixed. The ink so prepared is then ready for writing. This type of ink was still used in primary schools in some countries in Europe for some time after the Second World War.

## 3. Conclusions

The many uses and application of tannins described above need to be put into perspective with regards to possible further advances, existing drawbacks, and future potential. Of all the applications described above, leather tanning is still the main industrial use of vegetable tannins. While their use for this application has been progressively decreasing and limited to heavy duty leathers, as displaced for finer applications by chrome tanning, the real or perceived toxicity of chrome has spurred considerable research on feasible alternatives. Not all of these have gone in the direction of vegetable tanning, as synthetic resins such as melamine-based ones have been considered. However, vegetable tanning has regained some interest for soft leathers either in combination with oils or with synthetic resins. Although further development in this application for vegetable tannins cannot be considered as static, nonetheless the potential for future expansion is rather limited.

The second most important application of vegetable tannins is in wood adhesives. The strong shift away from synthetic resins based on formaldehyde has favored the interest in the use of tannins as well as of other natural raw materials for this application. The tannin adhesive technology is definitely more advanced, and more used, than other bio-sourced materials, having proved itself industrially in several countries over a number of decades. Its drawback at present is that their present supply is limited. The potential world supply of tannins is really huge, what is lacking, however, is a marked increase in the factories extracting them. A few new tannin extraction factories have been created in the last decade, but competition with other bio-sourced materials already industrially available either as waste or obtained by other already existing production sources is rather intense at present.

Medical and pharmaceutical applications are one of the more interesting and active fields of research at present for the evaluation of vegetable tannins. While some pharmaceutical applications already exist, for further progress the results being developed in this field need to be proven in vivo, this being an important phase of development. It is difficult to say for what specific pharmaceutical uses tannin might be successfully adopted. The main drawback here is that the balance of properties favorable and unfavorable to each application have to be evaluated. It is nonetheless an application that is likely to further flourish in the future.

More established and fully functional is the use of tannins in the beverage industry, be it wine, beer, or fruit juices. Their use will expand with the expansion of these markets due to the expansion of the population, but not for different applications in the field.

Tannin based foams, be it the more developed phenolic–furanic type, isocyanate-derived polyurethanes, or the newer, less developed non-isocyanate poly(hydroxy)urethanes, is a fast moving research field for thermal and/or acoustic insulation, for hydroponics, and a number of other applications. A considerable amount of research is still going on in this field, and some industrial trials too, but all this has not as yet materialized in an industrial application.

Tannin-based antipollution flocculants and corrosion inhibitors have been developed quite a long time ago, in the late 1960′s and early 1970′s and used industrially for some time. After a period of having practically disappeared from the market they are regaining favor, both in research and industrially, due to the interest in substituting bio-sourced material for somewhat toxic or oil derived synthetic materials.

As regards the other applications, foundry sand binders is used but it is unlikely to increase in market share due to the competition of other more performant materials. The same is valid for drilling fluids. Corrugated cardboard adhesives are used in a few developing countries for a niche industrial market, namely the moisture proofing of starch-bonded corrugated cardboard boxes for fruit exports having to pass through humid conditions, such as in the tropics.

Very new is the development of adhesives to bind teflon to steel and aluminum. While the technology exists and has proven itself it seems that the moment for a bio-sourced adhesive for this application has not yet come, although patents on the subject have been created. The writer supposes that this technology might eventually be used industrially once environmental protection awareness becomes stronger, and stricter environmental protection rules may force its application.

The situation is the same for the hard plastics used as matrices for abrasive angle grinders, discs, and automotive brake pads. Only time will tell if these developments, some of them patented, will ever reach industrial use.

Epoxy resins based on tannins have been developed by a few groups, one of which is in direct contact with interested industries. It is likely that some industrial development will eventually arise from this line of research, although none is known to date.

All the other applications are all in the purely experimental phase, and it is difficult to see if they will ever develop further or not.

Finally, ferric inks, the main source of writing inks for several past centuries, is definitely out of interest as more performant materials exist today, and no further interest in them is apparent.
